# Pitavastatin in cardiometabolic disease: therapeutic profile

**DOI:** 10.1186/1475-2840-12-S1-S2

**Published:** 2013-05-30

**Authors:** Luis Masana

**Affiliations:** 1 Vascular Medicine and Metabolism Unit, University Hospital Sant Joan, IISPV, CIBERDEM, Rovira and Virgili University, Sant Llorenç, 21. 43201 -Reus, Spain

## Abstract

Statins effectively lower low-density lipoprotein-cholesterol (LDL-C) and reduce cardiovascular risk in people with dyslipidemia and cardiometabolic diseases such as Metabolic syndrome (MetS) or type 2 diabetes (T2D). In addition to elevated levels of LDL-C, people with these conditions often have other lipid-related risk factors, such as high levels of triglycerides, low levels of high-density lipoprotein-cholesterol (HDL-C), and a preponderance of highly atherogenic, small, dense low-density lipoprotein particles. The optimal management of dyslipidemia in people with MetS or T2D should therefore address each of these risk factors in addition to LDL-C. Although statins typically have similar effects on LDL-C levels, differences in chemical structure and pharmacokinetic profile can lead to variations in pleiotropic effects, adverse event profiles and drug-drug interactions. The choice of statin should therefore depend on the characteristics and needs of the individual patient. Compared with other statins, pitavastatin has distinct pharmacological features that translate into a broad range of actions on both apolipoprotein-B-containing and apolipoprotein-A-containing lipoproteins. Studies show that pitavastatin 1 to 4 mg is well tolerated and significantly improves LDL-C and triglyceride levels to a similar or greater degree than comparable doses of atorvastatin, simvastatin or pravastatin, irrespective of diabetic status. Moreover, whereas most statins show inconsistent effects on HDL-C levels, pitavastatin-treated patients routinely experience clinically significant elevations in HDL-C that are maintained and even increased over the long term. In addition to increasing high-density lipoprotein quantity, pitavastatin appears to improve high-density lipoprotein function and to slow the progression of atherosclerotic plaques by modifying high-density lipoprotein-related inflammation and oxidation, both of which are common in patients with MetS and T2D. When choosing a statin, it is important to note that patients with MetS have an increased risk of developing T2D and that some statins can exacerbate this risk via adverse effects on glucose regulation. Unlike many statins, pitavastatin appears to have a neutral and even beneficial effect on glucose regulation, making it a useful treatment option in this high-risk group of patients. Together with pitavastatin’s beneficial effects on the cardiometabolic lipid profile and its low potential for drug-drug interactions, this suggests that pitavastatin might be a useful lipid-lowering option for people with cardiometabolic disease.

## Introduction

Numerous clinical trials have demonstrated that statins effectively lower low-density lipoprotein-cholesterol (LDL-C) and reduce cardiovascular (CV) risk in people with dyslipidemia and Metabolic syndrome (MetS) or type 2 diabetes (T2D) [1,2]. A recent individual patient meta-analysis of 14 randomized clinical trials, for example, showed that a 1.0 mmol/l (38.6 mg/dl) reduction in LDL-C was associated with a significant 21% proportional reduction in major vascular events in people with T2D (0.79, 0.72 to 0.86; *P* <0.0001) and that the reduction was similar in people without T2D (0.79, 0.76 to 0.82; *P* <0.0001) [1]. Most international treatment guidelines recommend lowering LDL-C to <2.6 mmol/l (100 mg/dl) in patients with high CV risk and to <1.8 to 2.0 mmol/l (70 to 80 mg/dl) in those at very high CV risk such as T2D with associated risk factors and established CV disease [3-7]. Despite these guidelines, the second Lipid Treatment Assessment Project (LTAP-2) showed that the proportion of patients who failed to achieve their recommended LDL-C target ranged from 16 to 53% across nine countries. Possible reasons for this failure include underdiagnosis, poor choice of first-line therapy, inadequate starting doses/failure to uptitrate or use additional therapy, and poor persistence with medications due to cost, adverse events and/or drug–drug interactions (DDIs) [8]. If LDL-C-target attainment rates are to be improved, these problems must be avoided. It is therefore important to tailor the choice of first-line lipid-lowering agent according to a patient's individual clinical profile and therapeutic need.

Although LDL-C-lowering is important for the reduction of CV risk, studies have shown that the risk of CV events in patients that fully attain their recommended LDL-C-target is only reduced by about one-third [9], leaving substantial residual risk. In addition to elevated levels of LDL-C, people with MetS and T2D often have other lipid-related risk factors, such as high levels of triglycerides, low levels of high-density lipoprotein-cholesterol (HDL-C), and a preponderance of highly atherogenic, small, dense low-density lipoprotein particles [10-12]. The optimal management of dyslipidemia in people with these conditions should therefore address each of these risk factors in addition to LDL-C. However, further studies are required to fully understand the therapeutic benefits of the various lipid-lowering drugs for the reduction of residual risk and to better define nonlow-density lipoprotein treatment targets.

## Pitavastatin

Pitavastatin is a relatively new member of the statin family. Pitavastatin was first introduced in Japan in 2003 for the treatment of primary hyperlipidemia or mixed dyslipidemia and has since been licensed for use in 13 countries worldwide, including the USA, Japan, China, Germany and Spain. Pitavastatin has recently been approved for use in 20 additional countries, including the UK, Australia, and France, and is pending approval in a further 12 countries. Compared with other statins, pitavastatin has a unique structure that contributes to a number of pharmacological benefits, including increased systemic bioavailability [13], a high level of oral absorption [14,15] and potent effects on LDL-C and HDL-C [16-19]. This review will discuss the potential benefits for pitavastatin in the treatment of patients with MetS or T2D, focusing on its beneficial effects on the atherogenic lipid triad, its neutral effects on glycemic control and its reduced potential for DDIs.

## Pitavastatin reduces LDL-C in people with Metabolic syndrome or type 2 diabetes

Numerous clinical trials have shown that pitavastatin is well tolerated and beneficially modifies the lipid profile with a similar or greater efficacy to equivalent doses of atorvastatin, rosuvastatin, simvastatin and pravastatin in a wide range of patient subgroups [20-30], including those with MetS or T2D [23,24,31]. For example, the 16-week, randomized head-to-head PATROL trial (*n* = 302) showed that pitavastatin 2 mg/day reduced median LDL-C levels by 41% in patients with risk factors for coronary artery disease and elevated LDL-C levels (≥3.63 mmol/l; 140 mg/dl), an effect that was noninferior to atorvastatin 10 mg/day (44%) and rosuvastatin 2.5 mg/day (42%) [30].

Similarly, a pivotal phase III study in 857 European patients with hypercholesterolemia or mixed dyslipidemia showed that 12-week treatment with pitavastatin 2 mg/day reduced LDL-C by 39.0% compared with 35.0% with simvastatin 20 mg/day (*P* = 0.014), whereas reductions with pitavastatin 4 mg/day and simvastatin 40 mg/day were 44.0% and 42.8%, respectively (*P* = NS) [21]. A second study in a similar population (*n* = 821) reported that 12-week treatment with pitavastatin 2 mg/day reduced LDL-C by 37.9% compared with 37.8% with atorvastatin 10 mg/day (*P* = NS) (44.6% and 43.5%, respectively, with pitavastatin 4 mg/day and atorvastatin 20 mg/day; *P* = NS) [20]. Importantly, the majority of patients with hypercholesterolemia or mixed dyslipidemia achieved their recommended National Cholesterol Education Program ATP III [3] and European Atherosclerosis Society [5] LDL-C targets within 12 weeks of treatment [20,21,32].

The Japanese long-term prospective post-marketing surveillance LIVALO Effectiveness and Safety (LIVES) Study (*n* = 20,279) [33] and the JAPAN-ACS study [27] – a prospective, randomized, open-label study in patients with hypercholesterolemia and acute coronary syndrome (ACS) (*n* = 251) – showed that the LDL-C-lowering efficacy of pitavastatin was similar among patients with and without T2D (–27.3% vs. –29.7%, respectively, in the LIVES study, and –35.7% vs. 36.4% in the JAPAN-ACS study). Furthermore, a subgroup analysis of the 12-week, randomized, open-label CHIBA study (*n* = 53) showed that the percentage reduction from baseline in LDL-C was significantly greater with pitavastatin than with atorvastatin in patients with MetS (45.8% vs. 39.1%; *P* = 0.0495) [23]. The CHIBA study was carried out in a relatively small population and results should therefore be treated with caution. However, the authors suggest that this difference may be due to the relationship between statin efficacy and obesity. Whereas the LDL-C-lowering efficacy of atorvastatin was attenuated by increased waist circumference, body weight and BMI, pitavastatin’s efficacy was unaffected by obesity-related parameters. Together these results suggest that pitavastatin might be useful for the reduction of LDL-C in people with MetS or T2D, many of whom are overweight or obese.

## Pitavastatin increases HDL-C levels in people with Metabolic syndrome or type 2 diabetes

Patients with MetS or T2D typically have low levels of HDL-C in addition to high levels of LDL-C and triglycerides [10,11]. A subanalysis of the J-LIT study showed that a 2.6 mmol/l (10 mg/dl) increase in HDL-C was associated with a 34.9% reduction in the risk of coronary events in patients with T2D [34]. Moreover, studies have shown that increasing HDL-C levels using statins can significantly reduce the progression of atherosclerosis and reduce CV and cerebrovascular risk in people with dyslipidemia irrespective of LDL-C levels [35-39].

Although most statins increase HDL-C levels to some extent, efficacy varies from statin to statin and effects are not always consistent between trials [39-41]. For example, the VOYAGER study – a meta-analysis of data from 32,258 high-risk individuals in 37 randomized clinical trials – showed dose-dependent increases in HDL-C ranging from 5.5 to 7.9% with rosuvastatin 5 to 40 mg and from 4.2 to 5.3% with simvastatin 10 to 80 mg, whereas the increases observed with atorvastatin were inversely related to the dose, falling from 4.5% with 10 mg to 2.3% with 80 mg [40]. In contrast, pitavastatin-treated patients routinely experience clinically significant, reproducible elevations in HDL-C [22,42-44].

**Figure 1:**
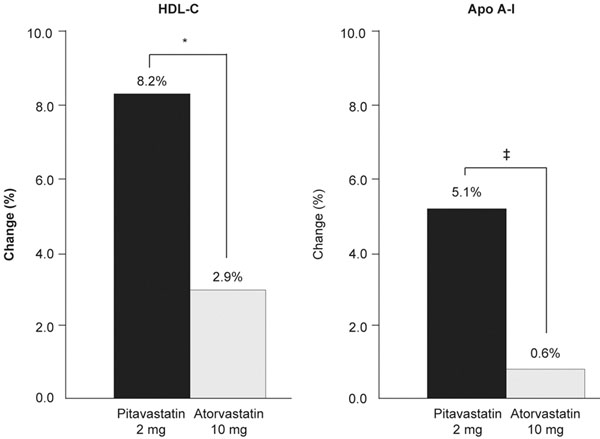
**Pitavastatin presents greater high-density lipoprotein-elevating and apolipoprotein A-1-elevating efficacy than atorvastatin.** Pitavastatin has more significant high-density lipoprotein (HDL)-elevating and apolipoprotein (Apo) A-1-elevating efficacy than atorvastatin in patients with hypercholesterolemia and glucose intolerance (52-week PIAT study). Values are means. *P = 0.031, two-sample *t* test. ^‡^*P* = 0.019, two-sample *t* test. Adapted from [24].

In the pivotal phase III studies, 12-week treatment with pitavastatin 2 to 4 mg/day increased HDL-C levels by 4 to 6% – an effect that was similar among patients treated with simvastatin and atorvastatin [20,21]. However, the longer-term (52 week) Effects of Pitavastatin and Atorvastatin on HDL-cholesterol Levels in Patients with Hyper-LDL Cholesterolemia and Glucose Intolerance (PIAT) study showed that pitavastatin 2 mg/day was associated with significant increases in HDL-C compared with atorvastatin 10 mg/day (8.2% vs. 2.9%; *P* = 0.031), an effect that was reflected by increasing levels of apolipoprotein A-1 (5.1% with pitavastatin vs. 0.6% with atorvastatin; *P*= 0.019) (Figure 1) [24].

In another long-term trial – the 70-month retrospective, single-center, observational CIRCLE study – HDL-C levels were increased by 13.4% with pitavastatin compared with only 7.0% with atorvastatin in patients with percutaneous coronary intervention (*n* = 743) (*P* = 0.029) [44]. These data suggest that the high-density lipoprotein (HDL)-elevating effect of pitavastatin might increase over time. Consistent with this observation, an extension of the pivotal phase III studies showed that pitavastatin-mediated elevations in HDL levels ultimately increased from 4% to 6% after 12 weeks to 14.3% after 60 weeks [22].

**Figure 2:**
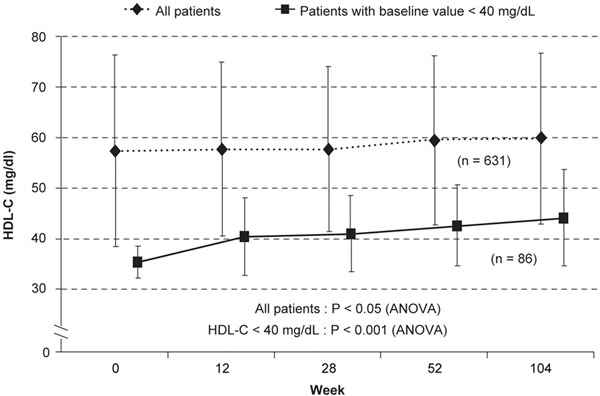
**Pitavastatin presents high-density lipoprotein-cholesterol-elevating efficacy that increases with time.** Pitavastatin has significant high-density lipoprotein-cholesterol (HDL-C)-elevating efficacy – especially in patients with low baseline HDL-C – that continues to increase over time (LIVES HDL substudy). Values are mean ± standard deviation. ANOVA, analysis of variance. Adapted from [46].

As for other statins [39], the degree of pitavastatin’s HDL-C-elevating efficacy appears to be related to serum concentrations of HDL-C at baseline. For example, the PATROL study showed that neither pitavastatin nor atorvastatin had a significant effect on HDL-C levels in patients with hypercholesterolemia and high baseline levels of HDL-C (~1.55 mmol/l; 60 mg/dl) [30]. In contrast, the KISHIMEN study in 178 Japanese subjects with hypercholesterolemia (58% with T2D) demonstrated significant pitavastatin-mediated elevations in HDL-C after 6 months ranging from 5.9% in the general cohort to 22.4% in patients with low baseline HDL-C (<1 mmol/l; 40 mg/dl) [45]. Similarly, the increase in HDL-C achieved with pitavastatin in the CIRCLE study was 21.3% among patients with a low baseline HDL-C level (≤1.17 mmol/l; 45 mg/dl) compared with 13.4% in the general population [44]. A subanalysis of the 2-year LIVES study showed that pitavastatin 1 to 4 mg/day significantly increased HDL-C levels by 5.9% in all subjects (*n* = 631) and by 24.6% (*P* <0.0001) in those with a low baseline HDL-C (<1 mmol/l; 40 mg/dl) (*n* = 86) (Figure 2) [46]. Moreover, HDL-C levels rose by 15.8% after patients with persistently low levels of HDL-C despite previous statin treatment switched to pitavastatin [42]. This observation suggests that patients might benefit from pitavastatin therapy if HDL-C remains unacceptably low on other treatments.

## Pitavastatin-mediated high-density lipoprotein elevation has the potential to reduce residual risk via a number of mechanisms

**Figure 3:**
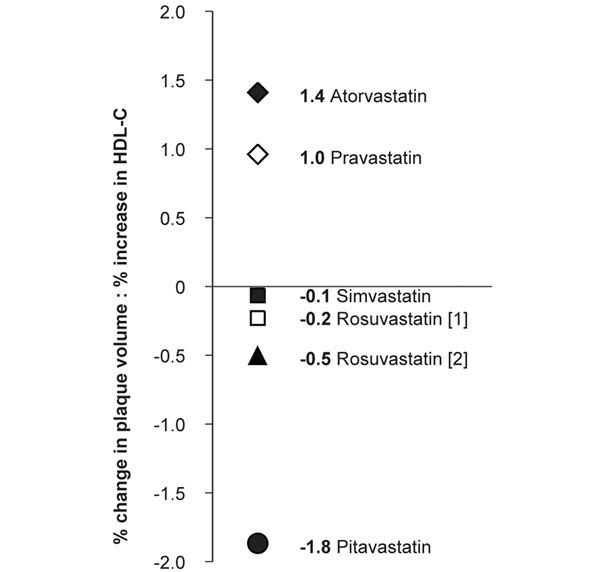
**Pitavastatin induced greater plaque volume reduction by high-density lipoprotein cholesterol unit increase.** Compared with other statins, pitavastatin is associated with the greatest reduction in plague volume per 1% increase in high-density lipoprotein-cholesterol (HDL-C) (KISHIMEN study). Adapted from [37].

HDL particles are central to the reverse cholesterol transport pathway, a process in which excess cholesterol is removed from peripheral cells and transported to the liver for excretion into bile [11]. The suggestion has therefore been made that elevations in HDL-C might slow the formation of atherosclerotic plaques and may reduce the residual CV risk by increasing the rate of cholesterol efflux from cells. A *post hoc* analysis of intravascular ultrasonography data from four prospective randomized clinical trials (*n* = 1455) showed that statin-treated patients with angiographic CHD experienced ≥5% reduction in the coronary atheroma volume when LDL-C levels were substantially decreased to <2.21 mmol/l (87.5 mg/dl) and HDL-C levels were increased by >7.5% [36]. However, a recent review of the literature suggests that, whilst the plaque volume change induced by a 1% reduction in LDL-C showed little difference using different statins (atorvastatin, pravastatin, pitavastatin, rosuvastatin, simvastatin), pitavastatin delivered the greatest reduction in plaque volume per 1% increase in HDL-C (Figure 3) [37]. Pitavastatin probably therefore beneficially modifies HDL function as well as quantity.

**Figure 4:**
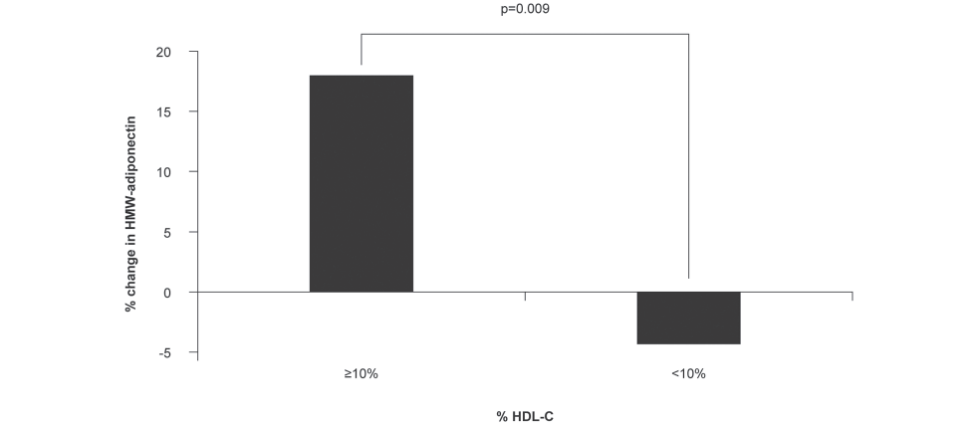
**Pitavastatin increases high-molecular-weight adiponectin levels.** In patients with Metabolic syndrome, the percentage change from baseline in high-molecular-weight (HMW) adiponectin levels is significantly greater in pitavastatin-treated patients achieving ≥10% increase in high-density lipoprotein-cholesterol (HDL-C) levels (PREMIUM study). Adapted from [51].

In addition to their role in reverse cholesterol transport, normal HDL particles can inhibit some of the atherogenic processes that occur in people with MetS and T2D, including increased oxidation [47-49], vascular inflammation [50,51], thrombosis [52], endothelial dysfunction [53], and reduced insulin sensitivity [54-56]. A 12-week, open-label multicenter study performed among 103 consecutive patients with hypercholesterolemia showed that patients with MetS (*n* = 69) had significantly higher mean levels of plasma high-sensitivity C-reactive protein and significantly lower mean levels of high-molecular-weight adiponectin than their counterparts without MetS (*n* = 34) [51]. In this study, a significant correlation was observed between baseline high-molecular-weight adiponectin levels and HDL-C values in patients with MetS (*r* = 0.318; *P* = 0.01) but not in those without. Moreover, an effectiveness analysis including 62 patients with MetS and 32 patients without showed that the level of high-sensitivity C-reactive protein was significantly decreased in MetS patients during pitavastatin treatment, whereas high-molecular-weight adiponectin levels did not change. When patients were grouped according to their percentage change in HDL-C, significantly greater pitavastatin-mediated increases in high-molecular-weight adiponectin were observed in patients with versus patients without HDL elevations ≥10% (*P* = 0.009) (Figure 4). This observation suggests that pitavastatin might slow the progression of coronary atheromas by modifying HDL-related effects on inflammation and oxidation, both of which are common in people with MetS and T2D.

## Pitavastatin has a neutral effect on glucose control

A number of clinical trials have highlighted a potential association between statin therapy and an increased risk of developing T2D [57-60]. For example, the Justification for the Use of Statins in Primary Prevention: An Intervention Trial Evaluating Rosuvastatin (JUPITER) study (*n* = 17,802) showed a significant 3.0% versus 2.4% increase in incident T2D among healthy adults treated with rosuvastatin 20 mg/day versus placebo for 1.9 years (*P* = 0.01) [61]. Similarly, a meta-analysis of 13 statin trials including 91,140 patients without T2D showed that statin therapy (atorvastatin 10 mg, pravastatin 40 mg, simvastatin 40 mg or rosuvastatin 20 mg) was associated with a 9% increased risk for T2D over 4 years (odds ratio = 1.09; 95% confidence interval = 1.02 to 1.17) [58].

The mechanisms by which statins might cause this effect are unclear. A recent study carried out in 27 patients with well-controlled T2D suggests that the potential diabetogenic effects of simvastatin and rosuvastatin are not driven by a detrimental effect on insulin sensitivity, but rather by a deterioration of insulin secretion [62]. In this study, patients were randomly assigned to receive either rosuvastatin 20 mg/day or simvastatin 20 mg/day for 6 months followed by the other treatment for a further 6 months. Both strategies were associated with a similar 0.8 to 0.9% increase in hemoglobin A1c levels after 12 months (*P* <0.001 vs. baseline for both) and similar trends in fasting plasma glucose levels. No changes in insulin sensitivity were detected throughout the study, whereas HOMAβ levels were significantly decreased in both groups.

**Figure 5:**
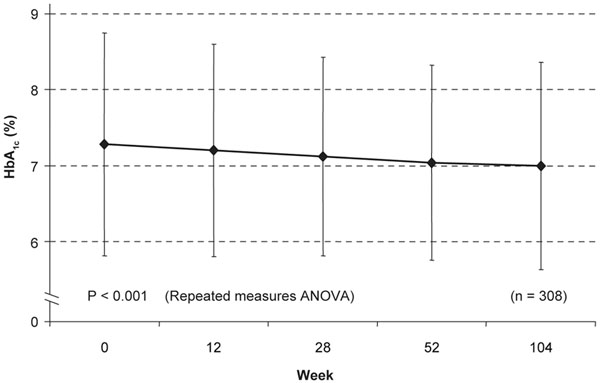
**Pitavastatin presents significant reductions in hemoglobin A1c levels that continue over time.** Pitavastatin is associated with significant reductions in hemoglobin A1c (HbA_1c_) levels that continue to decrease over time (LIVES study). Values are mean ± standard deviation. ANOVA, analysis of variance. Adapted from [46].

In contrast, the CAPITAIN study in 14 healthy male adults with well-defined MetS showed that 6-month treatment with the highest clinically available dose of pitavastatin (4 mg/day) did not significantly change mean glucose-related or insulin-related parameters, including fasting plasma glucose, the Homeostasis Model Assessment index, insulin levels, insulin/glucose ratios, or hemoglobin A1c levels, and showed that glycemic parameters were generally improved [63]. Consistent with these results, a subanalysis of LIVES study data showed a significant 0.28% decrease in hemoglobin A1c levels (*P* <0.001) among 308 patients with T2D after 2 years of pitavastatin treatment (Figure 5) [46]. These data suggest that whereas some statins are associated with adverse effects on glycemic control, pitavastatin has a neutral and possibly beneficial effect that is likely to be especially useful in people with, or at risk of developing, T2D – such as those with MetS. Definitive results on the impact of pitavastatin on the development of T2D are expected from the Japan Prevention Trial of Diabetes by Pitavastatin in Patients with Impaired Glucose Tolerance (J-PREDICT) study (*n*~ 1,240) in 2015 [64].

## Pitavastatin has a low potential for drug-drug interactions

People with MetS or T2D usually require multiple therapies for a range of CV risk factors. A study of >950,000 patient records from two US databases showed that 83% of patients with dyslipidemia used a CYP3A4-metabolized statin and that, of these, 25 to 30% also received a CYP3A4 inhibitor [65]. This suggests that patients treated with statins have a particularly high risk of developing DDIs, some of which may lead to drug discontinuations owing to adverse events. The best way to avoid this problem is to use a statin with a low potential for DDIs.

Whereas lovastatin, simvastatin and atorvastatin are metabolized mainly by CYP3A4 and fluvastatin and rosuvastatin are metabolized by CYP2C9, pitavastatin’s cyclopropyl group diverts the drug away from metabolism by CYP3A4 and allows only a small amount of clinically insignificant metabolism by CYP2C9. Moreover, studies in human hepatic microsomes have shown that, whereas the lactone metabolites of other statins are rapidly eliminated by CYP isoenzymes, both pitavastatin acid and lactone undergo limited metabolism [66]. It is not therefore surprising that the incidence of muscle-related adverse drug reactions identified during a *post hoc* analysis of the LIVES study was unaffected by the concomitant administration of pitavastatin with drugs known to inhibit a range of CYP isoenzymes [67]. These findings suggest that the pharmacokinetic data from interaction studies [68,69] are predictive of clinical experience and that pitavastatin has a low potential for DDIs. To date, the use of pitavastatin is contraindicated only in patients treated with cyclosporin or lopinavir/ritonavir combination therapy. Administration should be temporarily suspended in patients receiving erythromycin or fusidic acid, however, and the dosage should be limited to 2 mg in people treated with rifampicin. As for other statins, pitavastatin should be used with caution in people treated with fibrates or niacin.

Overall, studies demonstrate that statins are well tolerated and have similar effects on LDL-C levels in people with and without MetS or T2D. Compared with other statins, however, pitavastatin has a unique structure that contributes to a number of pharmacological benefits. These include potent LDL-C-lowering efficacy, clinically significant, reproducible elevations in HDL-C, a neutral or beneficial effect on glycemic control and a reduced potential for DDIs. Pitavastatin is therefore likely to be a useful treatment option for people with MetS or T2D.

## Abbreviations

CV: cardiovascular; DDI: drug–drug interaction; HDL: high-density lipoprotein; HDL-C: high-density lipoprotein-cholesterol; LDL-C: low-density lipoprotein-cholesterol; MetS: Metabolic syndrome;T2D: type 2 diabetes.

## Competing interests

LM is on the advisory boards of Amgen (GCAB), Sanofi, Danone, Esteve, and Recordati. LM has received lecture fees from MSD, Kowa, Danone, Esteve, Recordati, and Ferrer

## Declaration

This article has been published as part of *Cardiovascular Diabetology* Volume 12 Supplement 1, 2013: Statins in cardiometabolic disease: what makes pitavastatin different?The full contents of the supplement are available online at http://www.cardiab.com/supplements/12/S1. Publication of this supplement has been funded by Kowa Pharmaceutical Europe. Pitavastatin is a product marketed by the sponsor of the supplement. Articles are based on the proceedings of the World Congress for the Prevention of Diabetes. Authors received support with the preparation of their articles from GK Pharmacomm, an agency funded by the sponsor. All articles have undergone the journal’s standard peer review process. The Supplement Editors declare that they have no competing interests.
